# Therapeutic effects of bone marrow mesenchymal stem cells‐derived exosomes on osteoarthritis

**DOI:** 10.1111/jcmm.16860

**Published:** 2021-08-27

**Authors:** Yi Jin, Min Xu, Hai Zhu, Chen Dong, Juan Ji, Yake Liu, Aidong Deng, Zhifeng Gu

**Affiliations:** ^1^ Department of Rheumatology Affiliated Hospital of Nantong University Nantong China; ^2^ Medical School Nantong University Nantong China; ^3^ Research Center of Clinical Medicine Affiliated Hospital of Nantong University Nantong China; ^4^ Department of Orthopaedics The Affiliated Huai’an No. 1 People’s Hospital of Nanjing Medical University Huai’an China; ^5^ Department of Orthopaedics Affiliated Hospital of Nantong University Nantong China; ^6^ Department of Hand surgery Affiliated Hospital of Nantong University Nantong China

**Keywords:** exosomes, MEG‐3, MSCs, osteoarthritis

## Abstract

Mesenchymal stem cells (MSCs) have shown chondroprotective effects in clinical models of osteoarthritis (OA). However, effects of MSC‐derived exosomes on OA remain unclear. The study aimed to investigate the therapeutic potential of exosomes from human bone marrow MSCs (BM‐MSCs) in alleviating OA. The anterior cruciate ligament transection (ACLT) and destabilization of the medial meniscus (DMM) surgery were performed on the knee joints of a rat OA model, followed by intra‐articular injection of BM‐MSCs or their exosomes. In addition, BM‐MSC‐derived exosomes were administrated to primary human chondrocytes to observe the functional and molecular alterations. Both of BM‐MSCs and BM‐MSC‐derived exosomes alleviated cartilage destruction and subchondral bone remodelling in OA rat model. Administration of BM‐MSCs and exosomes could reduce joint damage and restore the trabecular bone volume fraction, trabecular number and connectivity density of OA rats. In addition, in vitro assays showed that BM‐MSCs‐exosomes could maintain the chondrocyte phenotype by increasing collagen type II synthesis and inhibiting IL‐1β–induced senescence and apoptosis. Furthermore, exosomal lncRNA MEG‐3 also reduced the senescence and apoptosis of chondrocytes induced by IL‐1β, indicating that lncRNA MEG‐3 might partially account the anti‐OA effects of BM‐MSC exosomes. The exosomes from BM‐MSCs exerted beneficial therapeutic effects on OA by reducing the senescence and apoptosis of chondrocytes, suggesting that MSC‐derived exosomes might provide a candidate therapy for OA treatment.

## 1 INTRODUCTION

Osteoarthritis (OA) is the most common type of chronic joint disease throughout the world, with the decline in living standards, functional disability and degenerative joint disorder.^1^, ^2^ Clinically, osteoarthritis (OA) is characterized by degradation of articular cartilage, inflammation of synovial and remodelling of subchondral bone.^3^, ^4^ OA patients suffer from severe pain, limitation of movement and a high risk of disability. Despite improvements in pharmacological, nonpharmacological and joint replacement surgery,^5^ the current treatments for osteoarthritis are a great challenge due to unsatisfactory overall effects and substantial adverse events.^6^ Knee arthroplasty removes the arthritic tissue but 10–20% of patients still report that pain remains after surgery.^7^ Although joint replacement surgery and knee arthroplasty temporarily alleviate the symptoms, overall results are still disappointing with the progression of OA.^8^ Therefore, it is of great significance to explore new therapeutic approaches against OA.

Mesenchymal stem/stromal cells (MSCs) are a population of multipotent stem cells, with the potential in adipogenesis, osteogenesis and chondrogenesis. Stem cell therapies exhibit great potential for the treatment of various diseases with its capability of tissue repair, immunomodulatory regulation and anti‐inflammatory effects.^9^ Interestingly, studies using cell tracking in cartilage repair show only limited cartilage formation by chondrogenic differentiation of the injected MSCs.^10^, ^11^ By contrast, rather than direct differentiation into the target tissue, accumulating evidence indicates that the biological and therapeutic effects of MSCs are mainly attributed to paracrine mechanisms mainly via secreting molecules, including growth factors, chemokines, cytokines and extracellular vesicles (EVs).^12^ Among these paracrine molecules, EVs may be the most valuable therapeutic factor.^13^

Extracellular vesicles, mainly made up of exosomes and micro‐vesicles (MVs), are now recognized to participate in cell‐to‐cell communication by transporting various proteins, microRNAs (miRNAs) and mRNAs.^14^ The therapeutic effects of exosomes for regenerative medicine have been thoroughly investigated. Because parent cell selection enhancing cargo specificity, avoiding of adverse immune responses or rejection seen in cellular therapies, exosomes continue to improve in regenerative medicine.^15^ Given the abundant contents and potential advantage as a carrier, the administration of MSCs‐EVs has provided a promising option in treating a great deal of benign or malignant diseases. Lung spheroid cell‐secretome (LSC‐Sec) and exosomes (LSC‐Exo) exhibited great therapeutic potential for lung regeneration in idiopathic pulmonary fibrosis (IPF).^16^ Better yet, exosomes derived from MSCs have facilitated immunosuppressive, pro‐angiogenic, anti‐apoptotic and anti‐fibrotic features in various tissues, including the skin,^17^ limbs,^18^ heart^18^ and lung.^15^ Recent a targeting peptide, cardiac homing peptide (CHP) is used to target intravenously infused exosomes to the infarcted heart, enhancing the targeting of exosomes.^19^ Furthermore, the role of exosomes as a regenerative medicine has potentially provided a novel nonsurgical, disease‐modifying approach for OA.^20^ Meanwhile, the contents in the exosomes could reduce MMP‐13 production in the joint space, which lead to the decreased cartilage destruction and the balance of cartilage synthesis. There are currently no therapeutic interventions that can reverse the process of OA. However, the exact roles of MSC‐derived exosomes in OA remain partially unclear. In this study, we aimed to explore the therapeutic effects and potential mechanism of MSCs‐exosomes on the OA animal.

## 2 MATERIALS AND METHODS

### 2.1 Animal model

The rats were housed in groups of 5 per plastic cage on sawdust bedding in a 12:12 light‐dark cycle (light‐on period, 6:00 AM–6:00 PM) with controlled temperature. They were fed a standard diet and were provided access to filtered water ad libitum. The animals were acclimatized for 1 week before the experiments. Cartilage destruction was evaluated using the Osteoarthritis Research Society International (OARSI) scoring system according to the percentage of the vertical clefts/erosion to the calcified cartilage.^21^ OA was induced unilaterally in the knee joint of donor animals by complete excision of the medial meniscus and resection of the anterior cruciate ligament. The knee joint of rats was injected with 100 µl exosome solution (100 µg) or MSCs (10^6^ cell) every week post‐surgery, which were sacrificed at the 8th week.

### 2.2 Isolation of bone marrow MSCs

Primary bone marrow MSCs (BMSCs) were incubated in low‐glucose Dulbecco's modified Eagle's medium (DMEM; Gibco, MD, USA) with 10% foetal bovine serum (FBS; Gibco) and 1% penicillin/streptomycin (Gibco). The cells were cultured in a humid incubator with 5% CO_2_. The initial cell number is 10^6^–10^7^/ml. After that, change the medium with L‐DMEM complete medium (10% FBS, L‐DMEM, 1% penicillin/streptomycin) every 3 days. Use an inverted microscope to observe its growth status every day. When the cells are cultured to 70%‐80% confluence of adherent cells, the cells are passaged.^22^ Cells at passage 3 were characterized for the following experiments.

### 2.3 Exosome isolation and procedures

This protocol of exosome isolation was based on previous exosome isolation method.^23^ Exosomes were isolated from conditioned medium of BM‐MSCs by ultracentrifuge, and almost 50ml conditional medium was collected. Initial spins consisted of 1000 *g*×10 min, 2000 *g* × 10 min and 10,000 *g* × 30 min. The supernatant was retained and spun at 100,000 *g* for 70 min, followed by resuspending in PBS. Then, the samples were conducted centrifugation at 100,000g for another 70 min. The final pellet was resuspended in 200 µl PBS and stored at −80℃. The protein concentration was detected by bicinchoninic acid (BCA) protein assay reagent (Pierce, Rockford, USA), and the concentration of exosomes is about 1 mg/ml.

### 2.4 Transmission electron microscopy of MSCs‐Exosomes

The morphology of MSCs‐exosomes was assessed by transmission electron microscopy (TEM). The exosome pellets were fixed in 3% (w/v) glutaraldehyde and 2% paraformaldehyde in cacodylate buffer and then loaded to copper grids coated with formvar. After washing, the grids were contrasted in 2% uranyl acetate. Following air drying, the samples were examined by TEM (Morgagni 268D, Philips, Holland).

### 2.5 Flow cytometry

The apoptosis assay was conducted according to the manufacturer's instructions of apoptosis detection kit (BD Biosciences, San Jose, CA). Cells were collected by trypsin and rinsed with PBS two times. Then, the samples were subsequently incubated with 5 μl Annexin V‐FITC/ PI for 15min in the dark, and thereby detected by using FACSCalibur flow cytometer (BD Biosciences, San Jose, CA).

### 2.6 Western blotting

The expression of protein was measured by Western blotting. Cells were lysed by radioimmunoprecipitation assay (RIPA) with 1 mM phenylmethanesulfonyl fluoride (PMSF, Beyotime, China). Bicinchoninic acid (BCA) protein assay reagent (Pierce, Rockford, USA) was used to determine the protein concentration of lysates. Equivalent amounts of protein were applied to SDS‐polyacrylamide gels for electrophoresis and transferred to PVDF membranes as described previously.^24^ Following blocking in 5% BSA solution, the membranes were incubated in primary antibody overnight at 4℃. After that, samples were treated with secondary antibody for 2 h at room temperature, followed by washing with PBS three times. At last, the samples were visualized using ECL (Millipore, USA).

### 2.7 Quantitative real‐time PCR

Total RNA was extracted from cells by using TRIzol (Invitrogen) according to the manufacturer's instructions. The first‐strand cDNA was synthesized by using reverse transcriptase Kit (TAKARA, Dalian, China). Quantitative real‐time PCR (RT‐qPCR) was conducted by SYBR Mix (Roche, Germany) on LightCycler^®^ 480 real‐time PCR machine (Roche, Germany). The relative values were quantified according to 2–ΔΔCT method. The sequences of the primers were listed as follows: ADAMTS5: F, GAACATCGACCAACTCTACTCCG; R, CAATGCCCACCGAACCATCT. MMP‐13: F, ACTGAGAGGCTCCGAGAAATG; R, GAACCCCGCATCTTGGCTT. COL2A1: TGGACGATCAGGCGAAACC; R, GCTGCGGATGCTCTCAATCT. GAPDH, F, GGAGCGAGATCCCTCCAAAAT; R, GGCTGTTGTCATACTTCTCATGG. MEG‐3: F, CTCCCCTTCTAGCGCTCACG; R, ‐CTAGCCGCCGTCTATACTACCGGCT.

### 2.8 Anterior cruciate ligament (ACL) transection and medial meniscectomy (MM)

The left knee was prepared in a surgically sterile fashion. Through mini‐arthrotomy, the anterior cruciate ligament (ACL) was transected with a scalpel, and MM was performed by excising the entire medial meniscus. The knee joint was irrigated, and the incision was closed. Ampicillin was administrated at the concentration of 50 mg/kg post‐surgery. The surgical site and the activities of the animals were observed daily. Six male Sprague‐Dawley rats (mean weight = 358 ± 5 g) underwent a unilateral sham operation as an additional control group.

### 2.9 SA‐β‐Gal staining

Senescence‐associated β‐galactosidase (SA‐β‐gal) staining was conducted to evaluate the senescence induced by IL‐1β according to the instruction. In brief, following exposure to IL‐1β, cells were washed in phosphate‐buffered saline (PBS) three times, followed by fixed in 4% paraformaldehyde for 15 min at room temperature. Then, the cells were incubated in SA‐β‐gal staining solution overnight at 37°C. The number of senescent cells was observed and counted under light microscope. The experiment was repeated independently three times.

### 2.10 Histological assessment

At 8th week post‐surgery, treatment‐related cartilage and bone changes in the ACLT‐joints were scored by two independent observers based on gross morphological appearance compared with the sham controls. Knee joints were fixed in 4% paraformaldehyde and decalcified in neutral 10% ethylenediaminetetraacetic acid (EDTA, PH 7.2) for 4 weeks at room temperature. Thereafter, the knees were embedded in paraffin blocks in the coronal position. Sections of 6 μ m thickness were performed for Safranin‐O and Fast‐Green staining. The articular cartilage was assessed using the Osteoarthritis Research Society International (OARSI) histological scoring system.^25^

After behavioural tests, the rats were sacrificed, and the gross morphologic changes in their right femoral condyle cartilage lesions were scored as follows: 0 = surface appears normal; 1 = minimal fibrillation or slight yellowish discoloration of the surface; 2 = erosion extending to the superficial or middle layers; 3 = erosion extending to the deep layer; and 4 = erosion extending to the subchondral bone.^26^ The right knee was routinely fixed with 4.0% paraformaldehyde at 4°C for 48 h and then decalcified with 10% EDTA at PH 7.4 for 4 weeks. After decalcification, each specimen was embedded in paraffin and sagittally sectioned through the medial femoral condyle at a thickness of 6 μm. The sections were then dewaxed in xylene and hydrated with graded ethanol series before being stained with haematoxylin and eosin (H&E).

### 2.11 Histological staining

The complete articulation of the knees was maintained in poly formaldehyde (4%) and was fixed for twenty‐four hours. Subsequent to traditional paraffin embedding, the articulation was cut into pieces at the thickness of 5 µm. The Safranin‐O‐Fast‐Green kit (ICH World, Woodstock, NY, USA) was used for staining. For histo‐morphometric assessment, area and depth of cartilage positive for Safranin‐O‐Fast‐Green were evaluated by Image‐Pro (Media Cybernetics, MD, USA). OA scores from the stained slides were determined using a modified Mankin's scoring system. The changes in subchondral bone osteoclasts TRAP and osteoblasts osteocalcin was measured by immunostaining method.

### 2.12 Bone parameter analyses

Surface reconstruction and three‐dimensional sagittal reconstruction of articular cartilage were assessed by mirco‐CT. Knee joints were dissected to carefully remove smooth tissues and scanned in a micro‐CT scanner SkyScan 1176 (Bruker, Belgium, 0.5 mm aluminium filter, 45 kV, 500 μA, resolution of 18 μm, 0.5° rotation angle). The scanning figures were reconstructed using NRecon software (Bruker, Belgium). The parameters include as follows: bone fraction volume (bone volume/total volume (BV/TV), reflecting the amount of bone; trabecular thickness (Tb. Th), describing the trabecular bone structure; trabecular number (Tb.N), explaining changes in bone mass; trabecular spacing (Tn. Sp) indicates average distance between trabecular bones; trabecular pattern factor(Tb. Pf) describes the connectivity between trabecular bones; SMI analyses the structural model parameters of the new bone; and trabecular connection(Conn. Dn) shows the number of trabecular connections per volume.^27^

### 2.13 Statistical analysis

Statistical analysis was performed using GraphPad Prism 5 (GraphPad Software, CA, USA). The data collected were expressed as mean ± standard deviation (SD). Statistical significance was assessed by Student's *t* test for comparing two groups and one‐way analysis of variance (ANOVA) for comparing multiple groups. A value of *p *< 0.05 was considered to evaluate statistical significance. All experiments were performed independently at least three times.

## 3 RESULTS

### 3.1 Characterization of BM‐MSC‐derived exosomes and the morphological effects on OA rats

**FIGURE 1:**
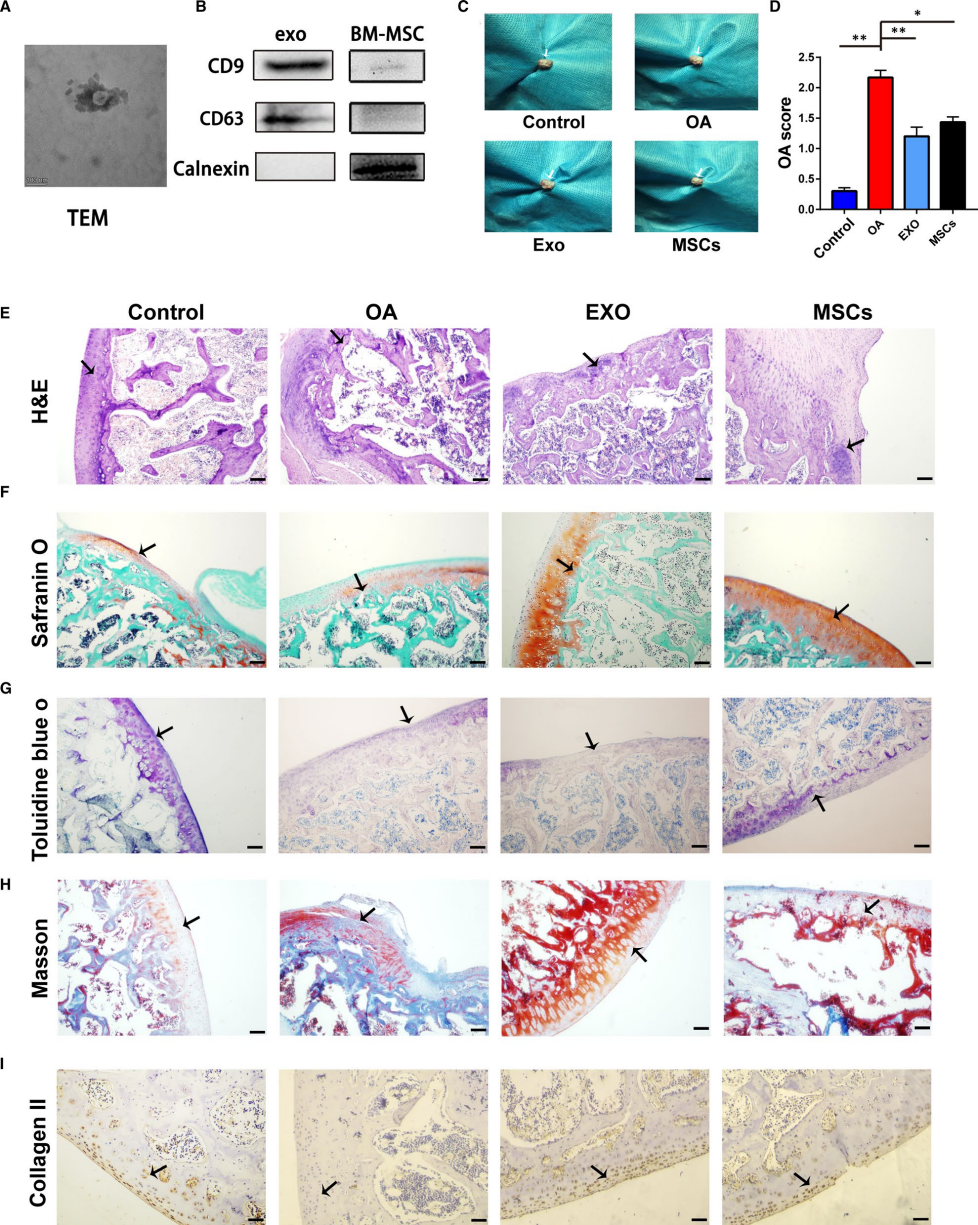
Effects of MSCs and their exosomes on morphological and histological alterations of knee joint in OA rats. A, Representative images of exosomes derived from BM‐MSCs by transmission electron microscopy. B, Identification of exosome by Western blotting. C, The morphological changes in knee joint after treatment of MSCs and exosomes. D, Semi‐quantitative OA score of each group. E–I, The histological evaluation of each group by H&E, Safranin O, toluidine blue O, Masson and IHC collagen II, respectively. Scale bar = 50 μm. TEM, transmission electron microscopy; H&E, haematoxylin and eosin; OA, osteoarthritis; EXO, exosome; MSCs, mesenchymal stem cells. * *p *< 0.05; **p < 0.01

The BM‐MSC‐derived exosomes were extracted from serum‐free medium by gradient centrifugation. The TEM showed that exosomes, with averaged diameter of 50–150 nm, presented the classic morphology as a vacuolar cup surrounded by a double membrane (Figure 1A). Western blotting further confirmed the positive expression of CD9 and CD63 with negative expression of calnexin in the total protein extracted from exosomes (Figure 1B). The OA rat model was constructed by surgical transaction of ACL and MM. Rats were injected with 100 µl exosomes solution (100 µg) or MSCs (10^6^ cell) every week post‐surgery, which were sacrificed at the 8th week. According to the morphological observation of the knee joint, yellowish viscous fluid and synovial membrane hyperplasia were observed in the articular cavity of the OA group, with cracks on the articular surface of the cartilage and osteophyte in the medial condyle of femur. At the same time, in the sham group, there was a small amount of colourless and transparent synovial fluid in the articular cavity, with smooth and complete cartilage articular surface. Osteophyte formation and synovial hyperplasia were not observed. Compared with the OA group, the articular cartilage surface of the exosomes group was still intact without the formation of osteophytes, and the fluid in the articular cavity was significantly reduced. The joint cavity condition of the MSCs group was also significantly better than that of the OA group. However, both of BM‐MSCs and their exosomes improved the status of the articular cavity, cartilage and femur in OA rats (Figure 1C). Semi‐quantification showed that score of the OA group was higher than that of control group, while the exosomes and MSC administration significantly reduced the OA score (Figure 1D).

### 3.2 Histological alterations of knee joint induced by MSCs and their exosomes

Histological staining was conducted to evaluate the effects of MSC‐EXO on the knee joints of OA model. According to H&E staining, OA group displayed disrupted and discontinuous cartilage with fibrous granulation tissue. However, the administration of MSCs or their exosomes obviously improved the condition of articular cartilage with less proliferative chondrocytes and clear hierarchical structure (Figure 1E). Based on the histological assessment by OARSI, treatment of MSCs and their exosomes could significantly decrease the OA grade. Similarly, the Safranin‐O‐Fast‐Green staining showed that the MSC and exosome groups had uniformly red staining of cartilage matrix and green staining of bone tissue with clear boundary (Figure 1F). Furthermore, MSC and exosome treatments increased the intensity of toluidine blue staining in cartilage matrix of OA rats (Figure 1G). Masson staining, mainly for collagen evaluation, demonstrated that MSC and exosome treatments increased positive area (red dye) and alleviate the destruction of articular cartilage surface (Figure 1H). Additionally, immunohistochemical staining of cartilage tissues showed that the intensity of collagen II expression in OA group significantly decreased compared with the control group, while the administration of MSCs and exosomes obviously recovered the expression of collagen II (Figure 1I). Mankin score was performed according to the structure, cell arrangement, matrix colouring, hydatid integrity and cartilage surface damage. Based on the staining above, MSCs and exosome groups had significantly lower Mankin score than that of the OA group (Fig. S1).

### 3.3 Effects of exosomes on degeneration of trabecular microarchitecture of subchondral bone in OA rats

**FIGURE 2:**
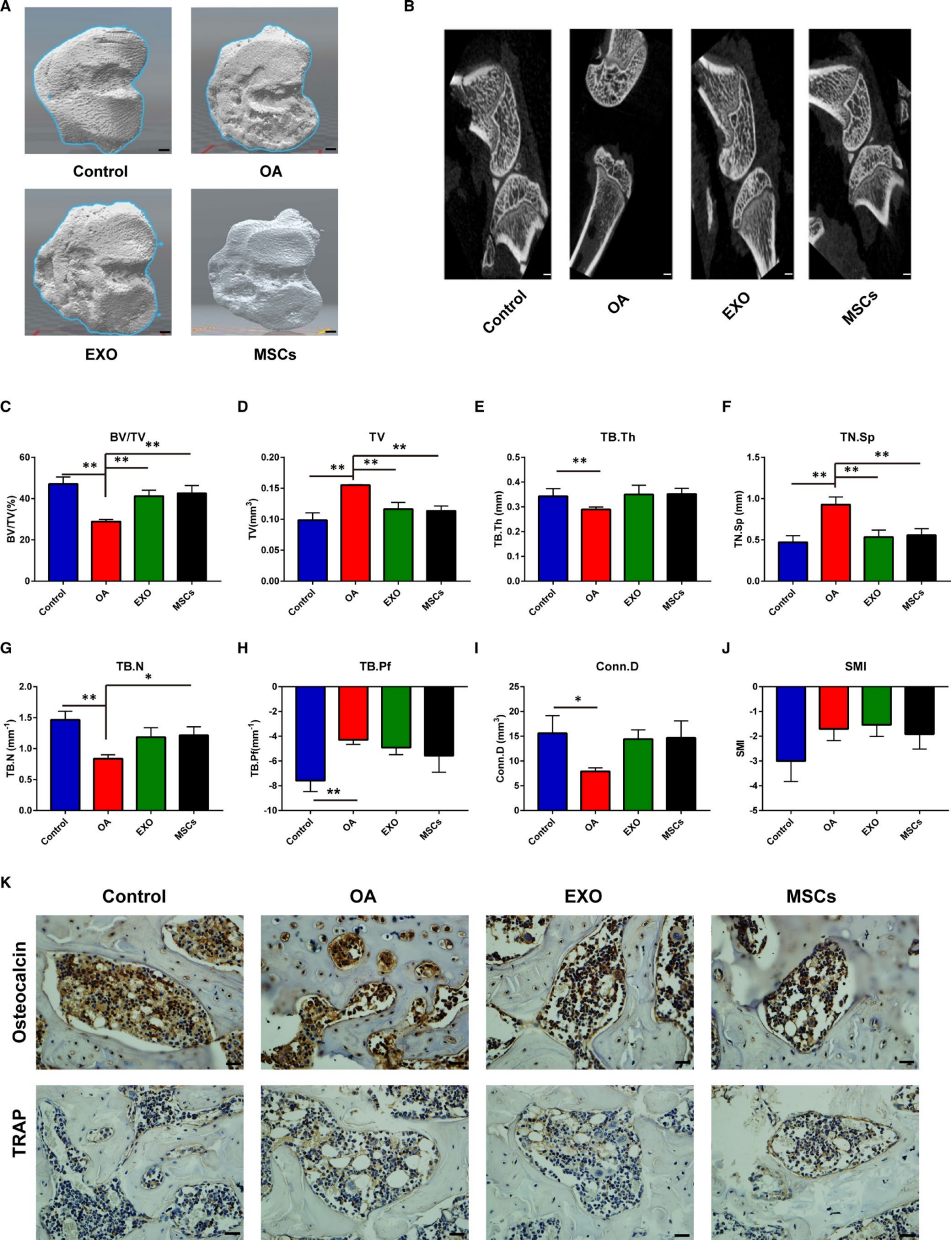
The MSC‐EXO alleviated the degeneration of trabecular microarchitecture of subchondral bone. A and B, Surface reconstruction and three‐dimensional reconstruction of articular cartilage by mirco‐CT. Scale bar = 1.0 mm. C–J, The OA subchondral bone parameters were calculated from the micro‐CT, including trabecular BV/TV, TV, TB. Th, TN. Sp, TB.N, TB. Pf, Conn.D and SMI. K, the immunohistochemical staining of osteocalcin and TRAP. Scale bar = 50 μm. OA, osteoarthritis; EXO, exosome; MSCs, mesenchymal stem cells. *p < 0.05; **p < 0.01

It is well‐known that degenerative changes in the subchondral region are accompanied by the degeneration in the articular cartilage. Transection of ACL and MM changed the biomechanical environment in the joint, thereby promoting the development of OA. Micro‐CT was conducted to investigate the changes in the subchondral bone. According to the observation of sagittal reconstruction, the trabeculae of OA subchondral bone presented sparse, broken and distorted morphology, with osteophytes at the joint edges. In contrast, MSC and exosome administration obviously improved the condition of bone trabeculae (Figure 2A,B). Furthermore, the trabecular bone volume fraction (BV/TV), trabecular number (TB.N.) and connectivity density (Conn.D) were all decreased in OA rats. Though no obvious alterations were observed in Conn.D and TB.N., MSCs and exosomes obviously elevated the BV/TV and TN. Sp. It suggested that BM‐MSCs and their exosomes might preserve the femoral subchondral bone microarchitecture in OA (Figure 2C–J). The expression of subchondral bone osteoblasts and osteoclasts was stained by immunohistochemistry to verify the changes in subchondral bone remodelling. In addition, the numbers of osteocalcin + positive osteoblasts in the OA group were significantly lower than that in the sham group, while the number of osteocalcin + positive osteoblasts in the Exo and MSC group was significantly higher than that in the OA group. Meanwhile, both of MSC and exosome treatments increased the osteocalcin staining intensity and decreased the TRAP staining, indicating the alterations in the population of osteoblasts and osteoclasts (Figure 2K).

### 3.4 Effects of MSCs‐exosomes on IL‐1β‐induced senescence and apoptosis in chondrocytes

**FIGURE 3:**
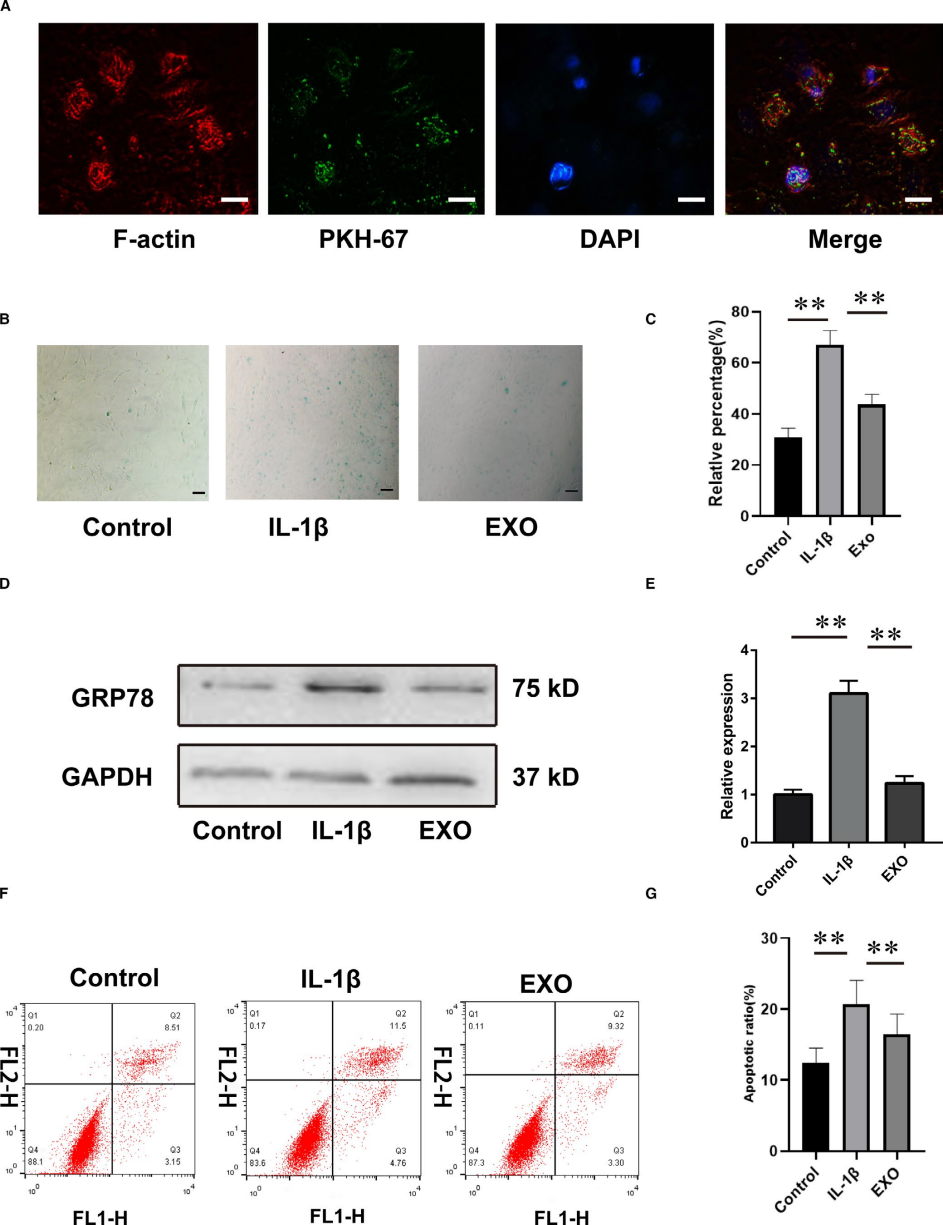
The MSCs‐exosomes alleviated the IL‐1β–induced senescence and apoptosis in chondrocytes. A, The uptake of exosomes in chondrocytes by PKH67 staining. Scale bar = 20 μm. B, β‐gal staining of chondrocytes in each group. Scale bar = 20 μm. C, Positive ratio of β‐gal staining in each group. D, The expression of GRP78 in each group by Western blotting. E, Relative expression of GRP78 in D. F, The apoptosis of chondrocytes detected by flow cytometry. G, The relative apoptotic ratio of chondrocytes. EXO, exosome. *p < 0.05; **p < 0.01

Based on the results of the OA model, we further discovered the effects of exosomes derived from BM‐MSCs on OA in vitro. Normal chondrocytes were treated with the IL‐1β to mimic the OA condition. Initially, we observed the uptake of BM‐MSCs‐exosomes by chondrocytes (Figure 3A). IL‐1β‐treated chondrocytes presented stronger β‐gal staining than that of control (*q* = 25.48, *p *< 0.001), while continuous treatment of exosomes partially reduced the positive ratio of β‐gal in the chondrocytes (*q* = 14.07, *p *< 0.001) (Figure 3B,C). Moreover, Western blotting demonstrated that BM‐MSCs derived exosomes also reversed GRP‐78 (senescence marker) expression induced by IL‐1β (Figure 3D,E). As shown in Figure 3F,G, the apoptotic ratio of chondrocytes in exosome‐treated group (11.93% ± 4.23%) was significantly lower (*q* = 6.867, *p *= 0.0068) than that in IL‐1β‐treated group (26.49% ± 4.48%). The evidence above indicated that MSC‐EXO alleviated senescence and apoptosis induced by IL‐1β in chondrocytes.

### 3.5 Molecular alterations induced by BM‐MSCs‐exosomes in chondrocytes

**FIGURE 4:**
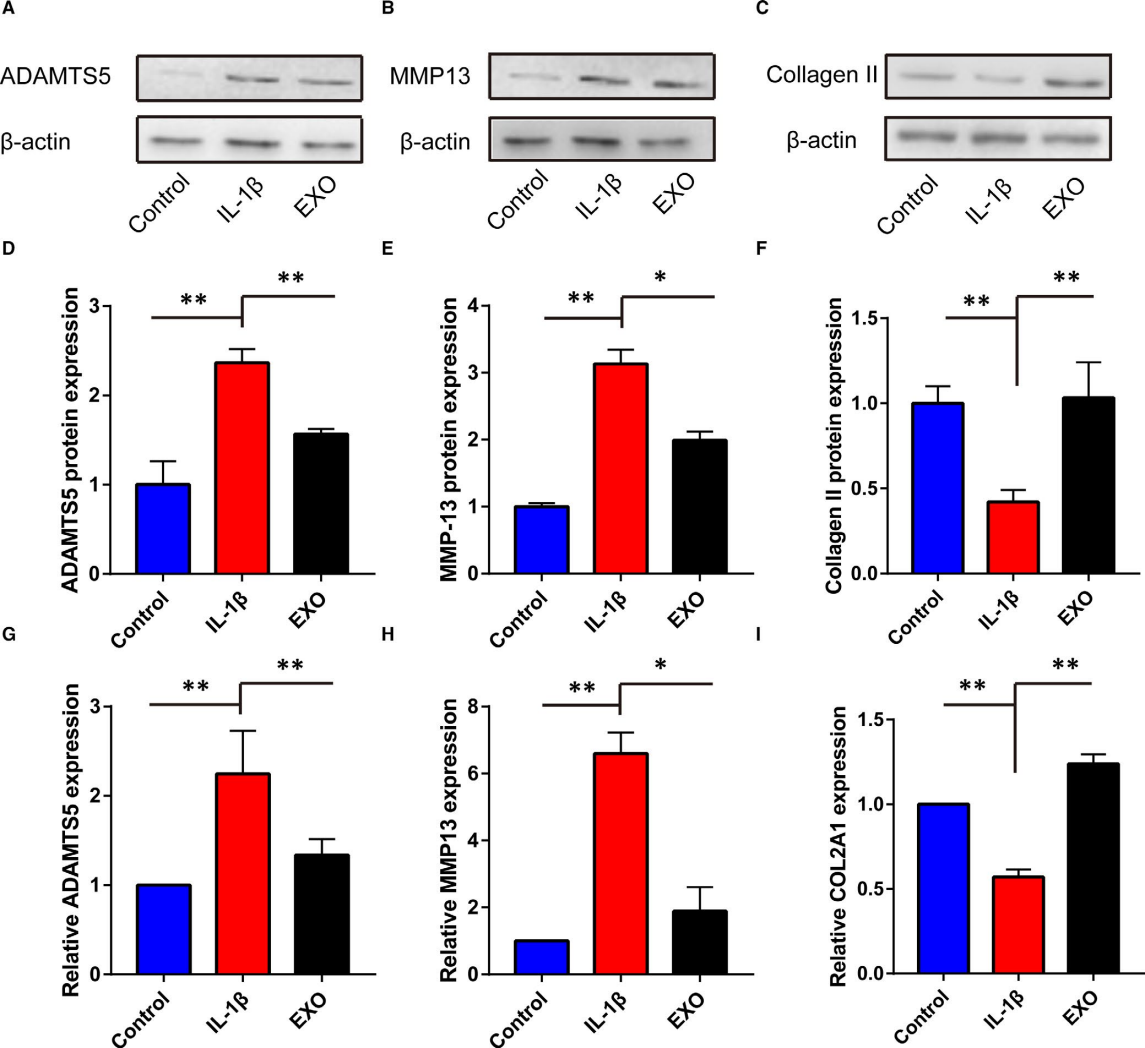
The BM‐MSCs‐exosomes mediated the molecular alterations in chondrocytes. A–C, The protein expression of ADAMTS5, MMP‐13 and collagen II in each group was detected by Western blotting. D–F, The relative expression of ADAMTS5, MMP‐13 and collagen II in A–C. G–I, The relative expression of ADAMTS5, MMP‐13 and COL2A1 in each group was detected by RT‐qPCR. EXO, exosome. *p < 0.05; **p < 0.01

Given the observation of biological behaviours, the molecular changes were explored following the administration of BM‐MSCs‐exosomes. Results of Western blotting demonstrated that IL‐1β significantly increased the expression of ADAMTS5 and MMP‐13 and decreased the expression of collagen II. However, the effects of IL‐1β on these proteins were abrogated by BM‐MSCs‐exosomes (Figure 4A‐F). In accordance, RT‐qPCR results showed that exosomes derived from BM‐MSCs could alleviate the alterations in ADAMTS5, MMP‐13 and COL2A1 induced by IL‐1β treatment (Figure 4G–I).

### 3.6 BM‐MSCs‐exosomes effected on chondrocytes through MEG‐3

**FIGURE 5:**
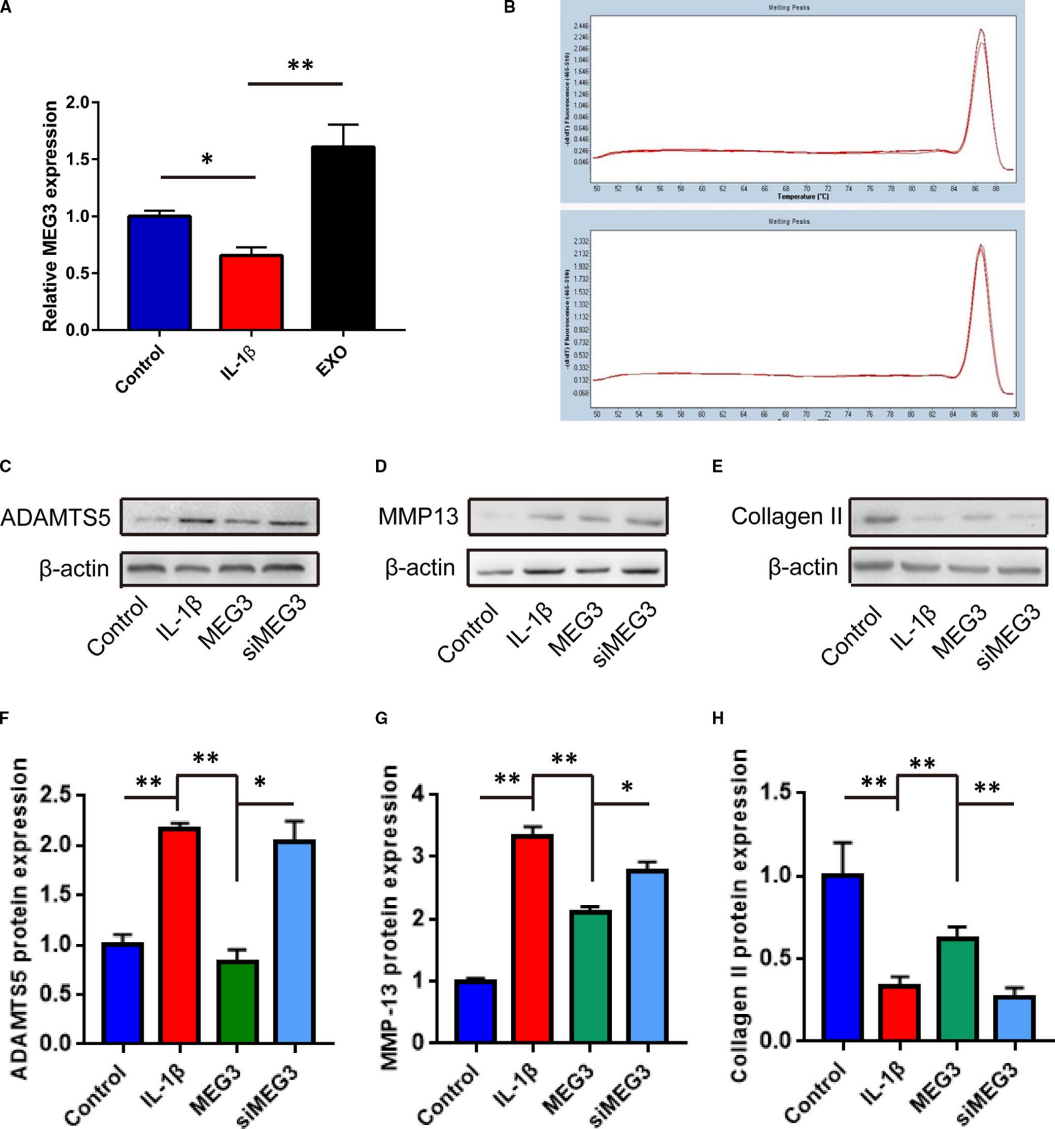
Exosomal MEG3 from BM‐MSCs induced molecular changes in chondrocytes. A, The expression of MEG‐3 in chondrocytes with IL‐1β or exosomes treatment detected by RT‐qPCR. B, The melt curves of MEG3 were observed by RT‐qPCR. (Figure 5B). C–E, The expression of MMP‐13, collagen II and ADAMTS5 was detected by Western blotting. F–H, The relative expression of markers in C–E. *p < 0.05; **p < 0.01

Various no‐coding RNAs are proved to play key roles in exosomes‐mediated biological functions. Initially, we found that IL‐1β treatment downregulated the expression of MEG‐3 (*q* = 4.81, *p *= 0.0334). However, further administration of BM‐MSC‐derived exosomes remarkably increased (*q* = 13.33, *p *= 0.0002) MEG‐3 expression (Figure 5A). Further, MEG‐3 was observed in melt curves of the exosomes derived from BM‐MSCs (Figure 5B). Following the treatment of IL‐1β, Western blotting confirmed that overexpressing MEG‐3 could upregulate the expression of MMP‐13 and collagen II with downregulation of ADAMTS5. Conversely, decreasing MMP‐13 and collagen II expressions and increasing ADAMTS5 expression were discovered in chondrocytes with knockdown of MEG‐3 (Figure 5C–H).

### 3.7 MEG‐3 inhibited IL‐1β‐induced senescence and apoptosis in chondrocytes

**FIGURE 6:**
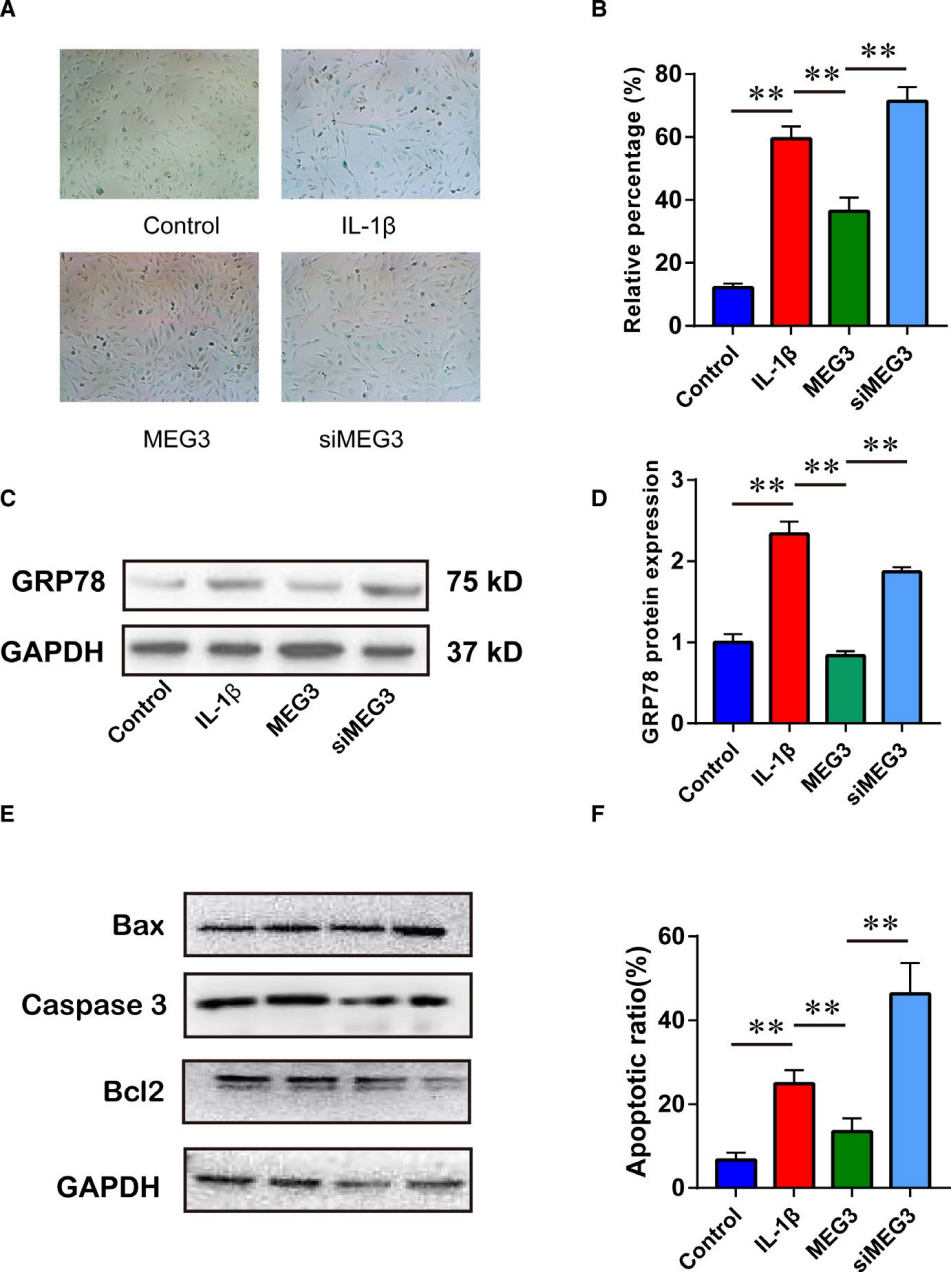
Exosomal MEG3 inhibited IL‐1β–induced senescence and apoptosis in chondrocytes. A, β‐gal staining of chondrocytes in each group. B, Positive ratio of β‐gal staining in each group. C, The expression of GRP78 in each group by Western blotting. D, Relative expression of GRP78. E, The apoptosis‐related protein of each group. F, The apoptosis of chondrocytes detected by flow cytometry. *p < 0.05;**p < 0.01

Evidence above showed effects of BM‐MSCs‐exosomes on chondrocytes could reverse IL‐1β–induced senescence and apoptosis. Thus, we continued to explore the effects of its content MEG‐3 on the chondrocytes. Overexpressing or knockdown MEG‐3 inhibited or increased the β‐gal staining intensity (Figure 6A,B) and GRP‐78 expression (Figure 6C,D), respectively. Moreover, MEG‐3 overexpression reduced the apoptotic chondrocytes induced by IL‐1β, while repressed MEG‐3 led to the increase in apoptotic cells (Figure 6E,F). These indicated that the exosomal MEG‐3 could regulated the IL‐1β–induced senescence and apoptosis in chondrocytes.

## 4 DISCUSSION

Osteoarthritis is a degenerative disease involved in various inflammatory processes. Nonsteroidal anti‐inflammatory drugs (NSAIDs) partially relieve OA symptoms. However, no obvious benefits are observed in the degeneration of cartilages and trabecular microarchitecture of subchondral bone. In addition, long‐term administration may lead to a range of serious side effects. With the capacity to alleviate the symptoms of osteoarthritis and cartilage damage, stem cell therapy is an emerging option for various diseases. Recent studies indicated that MSCs had shown therapeutic efficacy in OA animal model and clinical trials. MSC‐based tissue repair was first predicated on the hypothesis that these cells could differentiate into chondrocytes to replace the damaged tissue. However, it is now widely accepted that paracrine effects of MSC are a central mechanism of cell therapy promoting tissue regeneration, characterized by secretion of a broad spectrum of growth factors, chemokines, cytokines and EVs.^28^, ^29^ In the present study, we aimed to explore the therapeutic effects and underlying mechanisms of MSC‐derived exosomes on OA.

Previous studies indicated that EVs from MSCs could alleviate osteoarthritis through balancing synthesis and degradation of cartilage extracellular matrix and promoting chondrocyte proliferation. ACLT, a widely validated model, was performed in the current study to mimic OA features.^30^ Notably, administration of MSCs‐EXO significantly altered the key pathological features of OA, known as inflammation, catabolic matrix degradation and cellular apoptosis. In particular, they improved the status of the articular cavity, cartilage and femur in OA rats and alleviated the destruction of articular cartilage. OA progression is often accompanied by increased subchondral bone remodelling that enables mechanical forces to dynamically modify its structure. Thus, we further assessed effects of treatments on histo‐morphometric parameters of bone by micro‐CT. Bone formation is mainly caused by osteoblasts, and bone resorption is mainly caused by the activation of osteoclasts. Osteoblasts and osteoclasts constitute bone remodelling. We use immunostaining method to stain the changes in subchondral bone osteoclast TRAP and osteoblast osteocalcin. TRAP mainly reflects the changes in subchondral bone osteoclasts, and osteoclasts play a role in bone resorption; osteocalcin reflects the changes in subchondral bone osteoblasts, and osteoblasts play a role in bone formation.^31^ Bone remodelling is mainly the balance process of osteoblasts and osteoclasts. Once the balance is broken, the dynamic balance of bone remodelling will be broken, affecting bone remodelling. At the subchondral bone levels, higher bone volume and lower bone degradation were determined in MSCs or MSC‐EXO‐treated mice compared with OA controls. Accurate analyses of histo‐morphometric parameters of the entire articular cartilages and epiphyses suggested a chondroprotective role of BM‐MSCs and BM‐MSC‐derived exosomes. Besides, exosome treatment also significantly inhibited degeneration of trabecular microarchitecture of subchondral bone by mediating the ratio of osteoblasts and osteoclasts. Interestingly, exosomes shared similar anti‐OA effects against with MSCs. These results demonstrated that MSC‐Exo treatment was expected to replace MSC administration and plays a cartilage‐protective role in the in vivo OA model.

Furthermore, we constructed an in vitro OA model by culturing chondrocytes in IL‐1β‐contained medium to mimic inflammation environment in arthritis. IL‐1β plays a key role in the occurrence and development of OA, which was seemed to be associated with cartilage destruction.^32^ It is one of the most significant mediators of cartilage degeneration and joint inflammation.^33^ IL‐1β can stimulate chondrocytes to induce the expression of MMPs, ADAMTSs and other catabolic enzymes.^34^ Previous research has shown that aucubin treatment significantly reverses IL‐1β–induced cytotoxicity and attenuated the IL‐1β–induced chondrocyte apoptosis.^35^ Following the exposure to IL‐1β, continuous treatment of BM‐MSC‐Exo partially alleviated the senescence of chondrocytes, characterized by reduced β‐gal staining and GRP‐78 expression. In addition, MSC‐EXO administration remarkably inhibited the apoptosis of chondrocytes induced by IL‐1β treatment. Besides, the effects of IL‐1β on ADAMTS5, MMP‐13 and collagen II were abrogated by MSC‐Exo. The evidence above indicated that MSC‐Exo protected chondrocytes from senescence and apoptosis induced by IL‐1β. The exosomal biological contents are predominantly involved in cellular communication, structure and mechanics, immune modulation, metabolism and tissue regeneration. To elucidate the mechanism underlying the effects of MSC exosomes on anti‐OA and chondrocyte protection, we further examined the potential molecules in MSC‐derived exosomes that could elicit the observed functions.

Actually, previous studies tried to discover the biological contents in MSCs‐exosomes attributed to the therapeutic effects on OA. They found that MSC‐derived exosomes suppressed IL‐1β–induced apoptosis of chondrocytes and promoted cartilage repair in vivo by its content lncRNA‐KLF3‐AS1.^36^ Exosomal KLF3‐AS1 could act as a competitive endogenous RNA (ceRNA) by sponging miR‐206 to facilitate GIT1 expression, which led to chondrocyte proliferation induction, apoptosis inhibition and subsequent cartilage repair.^37^ Besides, exosomal Annexin A1 derived from Ad‐MSCs could serve as a therapeutic factor for OA by chondroprotective effects through activation of NF‐κB and activator protein‐1.^38^ Moreover, a recent study showed that exosomes from miR‐92a‐3p‐overexpressing MSCs enhanced chondrogenesis and ameliorated cartilage degradation by directly targeting Wnt5a. Indeed, previous studies indicated that lncRNA MEG‐3 regulated the proliferation, apoptosis and ECM degradation of chondrocytes through miR‐93/TGFBR2 axis or miR‐16/SMAD7 axis.^39^, ^40^ In the current study, we explored whether the lncRNA MEG‐3 in the exosomes played an important role in the therapeutic functions for OA. The expression of lncRNA MEG‐3 was altered with the IL‐1β treatment and exosomes derived from BM‐MSCs. Further, we conducted a series of functional and molecular experiments to investigate the role of lncRNA MEG‐3. Overexpressing or downregulating MEG‐3 could regulate the expression of chondrocyte‐related markers MMP‐13, collagen II and ADAMTS5, respectively, which were previously mediated by IL‐1β administration. Furthermore, MEG‐3 could alleviate the senescence and apoptosis induced by IL‐1β in the chondrocytes. These observations were consistent with the evidence above with exosomes treatment, suggesting exosomal lncRNA MEG‐3 might partially account for the BM‐MSCs‐exosome‐mediated therapeutic effects on OA.^3^

## 5 CONCLUSIONS

In summary, based on the *in vitro* and *in vivo OA* model, our study demonstrated that BM‐MSC–derived exosomes are a candidate cell‐free therapy for the treatment of OA. In addition, the lncRNA MEG‐3 might account for the anti‐OA effects mediated by MSC exosomes. Though the current evidence is promising, more investigations should be conducted to further validate the underlying mechanisms.

## CONFLICT OF INTEREST

The authors declare that the research was conducted in the absence of any commercial or financial relationships that could be construed as a potential conflict of interest.

## AUTHOR CONTRIBUTION

**Yi Jin:** Data curation (equal); Methodology (equal); Writing‐original draft (equal); Writing‐review & editing (equal). **Min Xu:** Methodology (equal); Software (equal); Writing‐review & editing (equal). **Hai Zhu:** Data curation (equal); Methodology (equal); Writing‐original draft (equal); Writing‐review & editing (equal). **Chen Dong:** Software (supporting); Validation (supporting). **Juan Ji:** Resources (supporting); Visualization (supporting). **Yake Liu:** Conceptualization (equal); Formal analysis (equal). **Aidong Deng:** Conceptualization (lead); Funding acquisition (lead). **Zhifeng Gu:** Conceptualization (equal); Funding acquisition (lead).

## Supporting information

Supplementary Material

## Data Availability

The raw data supporting the conclusions of this article will be made available from the corresponding author on reasonable request.
