# Supplementary material for: Therapeutic effects of bone marrow mesenchymal stem cells‐derived exosomes on osteoarthritis
Source: J Cell Mol Med. 2021 Aug 27;25(19):9281–94. doi: 10.1111/jcmm.16860 (PMC8500984; doi:10.1111/jcmm.16860)
Supplement: Supplementary file 1 — Supplementary Material [file JCMM-25-9281-s001.docx]

Supplementary Material

## Supplementary Figures


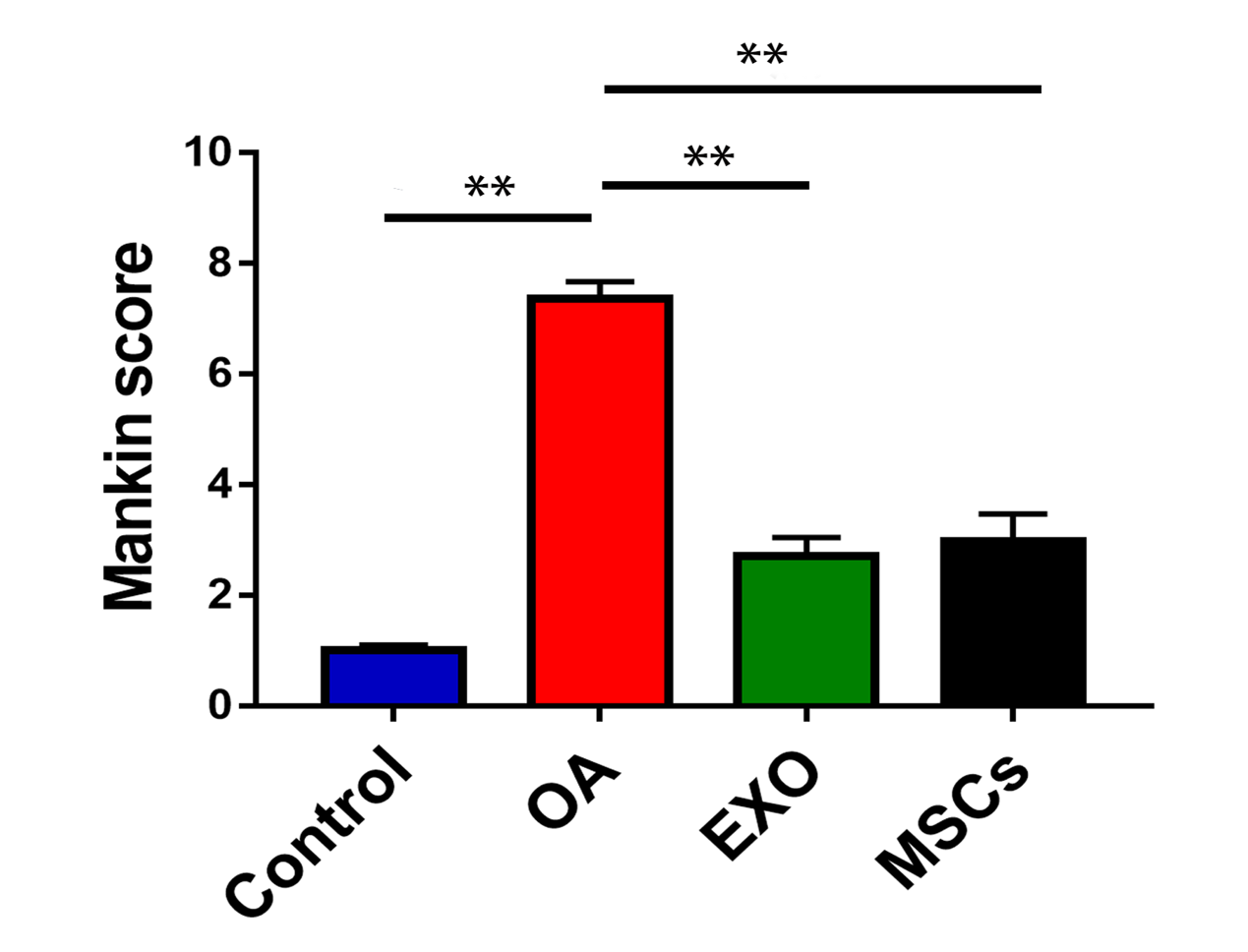


**Supplementary Figure 1.** **Mankin score of each group.** At the 8^th^ week after the operation, rats were graded according to the structure, cell arrangement, matrix coloring, hydatid integrity, and cartilage surface damage in the Mankin method. OA, osteoarthritis; EXO, exosome; MSCs, Mesenchymal stem cells. ^*^*P*＜0.05; ^**^*P*＜0.01.
